# Susceptibility to COVID-19 Nutrition Misinformation and Eating Behavior Change during Lockdowns: An International Web-Based Survey

**DOI:** 10.3390/nu15020451

**Published:** 2023-01-14

**Authors:** Maria A. Ruani, Michael J. Reiss

**Affiliations:** Curriculum, Pedagogy and Assessment, IOE, UCL’s Faculty of Education and Society, University College London, London WC1E 0ALT, UK

**Keywords:** nutrition misinformation, eating behavior change, dietary change, sources of information, information-seeking behaviors, public health, communication, COVID-19 infodemic, social media, misinformed beliefs

## Abstract

To understand the susceptibility to nutrition-health misinformation related to preventing, treating, or mitigating the risk of COVID-19 during the initial lockdowns around the world, the present international web-based survey study (15 April–15 May 2020) gauged participants’ (*n* = 3707) level of nutrition-health misinformation discernment by presenting them with 25 statements (including unfounded or unproven claims circulated at the time), alongside the influence of information sources of varying quality on the frequency of changes in their eating behavior and the extent of misinformation held, depending on the source used for such changes. Results revealed widespread misinformation about food, eating, and health practices related to COVID-19, with the 25 statements put to participants receiving up to 43% misinformed answers (e.g., *‘It is safe to eat fruits and vegetables that have been washed with soap or diluted bleach’*). Whereas higher quality information sources (nutrition scientists, nutrition professionals) had the biggest influence on eating behavior change, we found greater misinformation susceptibility when relying on poor quality sources for changing diet. Appropriate discernment of misinformation was weakest amongst participants who more frequently changed their eating behavior because of information from poor quality sources, suggesting disparities in the health risks/safety of the changes performed.

## 1. Introduction

The early period of the COVID-19 pandemic was widely seen as a terrifying period of time because of distressing uncertainties and limited knowledge about transmission and protection, with social fear, panic, and anxiety being exacerbated by misinformation and disinformation exposure [1,2,3]. Indeed, this time was characterized by little to an inexistent understanding about contagion odds via food, packaging, surfaces and air, virus survival time, viral load, prevention, treatment, severity, duration, and the role of diet, food, and nutrition. Because of this, a storm of speculations and assumptions were made and propagated prematurely, many of which were unfounded, unproven, or discredited. Amid warnings by the World Health Organization (WHO) about falsified diagnostics and therapeutics for the novel coronavirus [4], baseless (and often unsafe) food and health approaches were widely disseminated through various channels of information [5,6], most alarmingly by national presidents, including Donald Trump in the United States, who alluded to bleach and sunlight as preventative methods [7] and Alberto Fernández in Argentina, who recommended hot drinks to kill the virus [8]. Thus, nutrition misinformation susceptibility, discernment, and subsequent eating behavior changes are the focus of the present research study.

Undoubtedly, health and nutrition misinformation and disinformation have been a major public health concern since the outset of the COVID-19 pandemic [6,9,10], to the extent that the ‘infodemic’ phenomenon was deemed a secondary public health threat by the WHO [11]. Infodemics research captures the augmented fast spread and virality of health misinformation and disinformation in an attempt to reduce exposure (e.g., via takedowns) or to mitigate impact once exposed (e.g., through debunking). In the absence of a healthy information environment, ‘inoculation’ theory (pre-bunking) aims to find ways to preemptively ‘immunize’ individuals before exposure by helping to generate their own misinformation ‘antibodies’ to boost misinformation discernment and reduce ‘infection’ susceptibility [12]. Once ‘infected’ (accepting misinformation as true), the risks of forming or reinforcing misinformed beliefs about health and nutrition are many—from making misled decisions that could result in health harm such as gargling bleach [13] or ingesting methanol [14], to falling for a false sense of security at the expense of not complying with official safety guidelines, experiencing a reduced intention to partake in preventative health behaviors, engaging in unnecessary monetary expenditure, and passing on misinformation to others.

Numerous international reviews have assessed the sources of health misinformation propagation, with the highest proportion of health misinformation spread through social media platforms (most prominently Facebook, followed by WhatsApp instant messages), popular personalities, and mainstream media [1,15]. However, the extent of COVID-19 nutrition and health misinformation held by individuals has been less clear. Additionally, while there were vast amounts of available data about food and eating behaviors at the outset of the COVID-19 pandemic, no study has yet looked at misinformation susceptibility based on information sources of varying quality used for dietary change.

In an effort to understand the susceptibility to nutrition and health misinformation related to preventing, treating, or mitigating the risk of COVID-19 soon after it was declared a global pandemic, the present international web-based survey study was timed to take place during the initial 2020 lockdowns and designed to gauge the respondents’ level of nutrition and health misinformation discernment during this period, alongside the influence of different information sources on the frequency of changes in eating behavior. On this premise, the present survey study attempts to explore:

- The respondents’ views about certain substances, foods, nutrients, dietary approaches, and health practices in relation to the transmission, infection, protection, or treatment of COVID-19 (i.e., nutrition-health misinformation discernment).
- The self-reported influence of different interpersonal and generic sources of diet and nutrition information or advice on the frequency of changes in food and eating behaviors (i.e., reliance on an information source for making dietary changes).
- Trends in online information seeking and dietary change frequency amongst respondents whose interest in nutrition increased since the pandemic, compared to those whose interest did not increase (i.e., interest in nutrition, online information seeking, and dietary changes).
- The relationship between the use of information sources of varying quality for changing eating behavior and the proportion of misinformed views about food, diet, nutrition, and health practices concerning COVID-19 during the first-wave of lockdowns around the world (i.e., nutrition-health misinformation susceptibility when relying on lower versus higher quality sources for changing diet).

## 2. Materials and Methods

### 2.1. Study Design and Population

Survey responses were collected between 15 April and 15 May 2020. This period corresponds to the first strict pandemic lockdowns unprecedently and simultaneously taking place in more than 90 countries around world [16]. This was in the context of a much feared [17], fast-spreading [18], and little-known version of a novel coronavirus associated with severe acute respiratory symptoms and an initially suspected mortality rate above 3% [19], at a time when no diagnostic tools, no vaccines, and no therapeutics of any kind were yet developed [20].

The adoption of a non-probabilistic, opportunity sampling method enabled a rapid distribution of the survey questionnaire among over 100,000 email subscribers of The Health Sciences Academy, who represent a population [21] with an above-average education and an interest in nutritional matters predominantly from the United Kingdom, the United States, and Australia (among circa 170 other countries), and the gathering of a relatively large number of voluntary responses in a short period. Speed of data collection was critical at a time in global history when knowledge about COVID-19 was scarce yet precipitously being updated amid a constant back-and-forth of conjectures and disinformation [22]. The utilization of a survey for this type of exploratory research has been reported to provide an advantage in this respect [23,24], notwithstanding its known limitations [25].

A total of 3781 participants answered survey questions. In total, 22 minors (participants aged 17 or below) were excluded from the analysis. Out of the 3759 participating adults, 3707 provided consent for their answers to be used for anonymized analysis. Therefore, the final sample consisted of 3707 respondents.

### 2.2. Ethical Considerations

Ethical approval was obtained from the Research Ethics Committee at the University College London, with the reference number Z6364106/2018/06/67. All aspects of the survey design, distribution, and data treatment complied with this approval.

Participation was optional, with no known risks for participating or not participating and with no associated compensation, financial incentive, or direct benefit. Consent was requested on the survey front page before accessing the rest of the questionnaire. All responses were captured online through a web-based form constructed on the SurveyMonkey platform, and participants were given the option to skip questions and to exit the questionnaire at any time without consequences. Survey design settings were adjusted upfront to restrict participants from taking the survey more than once and to collect responses without participants having to formally submit their form. The survey was administered via a web link included in the invitation email and could be completed from any computer or device with a browser and Internet connection.

### 2.3. Instrument Measures and Outcomes

The survey instrument developed for this research study was a questionnaire in English, consisting of a voluntary consent question on the front page, followed by 13 questions (many with sub-questions) in three main parts, as described below.

#### 2.3.1. General Demographics and Characteristics

This first part collected participant data on country of residence, employment status, age, gender, number of physical and digital books owned, current diet, self-reported health status, interest in nutrition since the COVID-19 outbreak, and nutrition profession involvement or lack thereof.

Supplementary Table S1 describes the socio-demographic characteristics of respondents, with a predominance of women (78.5%), which is likely a reflection of the general demographics of The Health Sciences Academy and the general tendency for females to be substantially more likely than males to participate in fully voluntary online surveys, including those related to nutrition and health [26,27,28]. A total of 124 countries worldwide were represented, with 40.3% of respondents being from the United Kingdom, 14.5% from the United States, 8.7% from India, 4.9% from Canada, 4% from Australia, 2.6% from Ireland, and 25.3% from another 118 countries. Adults aged 18 to 70 or older were included in the sample, with around two-thirds of them being 26 to 55 of years of age (70.9%) and employed (65.6%). More than half of respondents reported owning over 100 books (51.5%) and 16% of them more than 500 books. When asked about their current diet, the majority stated they consume plant foods (99.7%) in different proportions, most commonly a balance of plant foods and animal foods (48.9%). The respondents’ self-reported health was mostly good or better (36.1% ‘great’ and 47.7% ‘good’). Respondents were also asked whether their interest in nutrition had increased since the COVID-19 outbreak, and nearly 6 in 10 (56.1%) said ‘yes’. Lastly, although just over half (51.1%) had no involvement in the nutrition profession, 23.4% affirmed being a nutrition professional, and 25.6% said they were studying for this. Additional participants’ characteristics are detailed in Supplementary Table S1.

#### 2.3.2. Views about Food, Eating, and Health Practices Related to COVID-19

The second part of the survey consisted of 25 statements related to certain substances, foods, nutrients, dietary approaches, and health practices in relation to the transmission, infection, prevention, or treatment of COVID-19 (Supplementary Table S2) that could be marked as ‘correct’, incorrect’, or ‘not sure’ by respondents. The presented order of the statements was randomly rotated/randomized for each respondent.

Because no published research studies evaluating commonly disseminated COVID-19 nutrition misinformation were available at the time, 21 out of the 25 statements in this part of the survey were written based on unfounded or unproven claims and assumptions regarding certain substances, foods, nutrients, and diets for COVID-19 gathered from multiple sources of information since the global outbreak, including but not limited to WhatsApp messages forwarded numerous times, social media (e.g., Facebook, Twitter, YouTube), news outlets, TV and radio broadcasts, online websites, Internet searches, and misinformation warnings issued by government agencies (UNICEF, WHO, etc.). These statements were written by a nutrition science educator to correspond closely to the information from the original sources and went through a number of verification and fine-tuning cycles with input from a professor in science education.

#### 2.3.3. Eating Behavior Change Frequency per Information Source

The third part of the survey was designed to measure respondents’ self-reported frequency of changes in food and eating practices since the COVID-19 outbreak based on nutrition information or dietary advice from 22 possible sources (Supplementary Table S3):

- Eleven of these sources were interpersonal (individuals such as one’s medical doctor or GP, nutrition professionals, government officials, influencers followed on social media, family members, friends, colleagues, or peers).
- The other 11 were general sources of nutrition information or dietary advice (such as Google or Internet searches, Facebook, Twitter, and other social media, WhatsApp, Viber, Messenger, or other kinds of private messages, WHO, UNICEF, CDC, NHS, or other official government websites).
- Possible sources were listed in a randomly rotated order for each participant within a matrix-style question asking: *“Since the COVID-19 outbreak, have you changed the way you eat or what you eat based on nutrition information/dietary advice from the following sources?”*

The frequency of dietary behavior changes for each of the listed sources of nutrition information or dietary advice was assessed using the scale ‘never’, ‘hardly’, ‘occasionally’, ‘repeatedly’, and ‘continually’.

### 2.4. Statistical Analysis

The statistical analysis of the results was conducted using Python software (Version 3.7.0; Python Software Foundation, Delaware, U.S.) and Microsoft^®^ Excel^®^ for Microsoft 365 MSO (Version 2202, Build 16.0.14931.20118, 32-bit).

For the initial analysis of the collected data, descriptive statistics were applied, such as computing numbers of responses (# observations), percentages, mean values, and standard deviations. For cross-tabulations comparing survey answer choices among different groups of respondents, *p* values, effect sizes (Cohen’s d), and weighted averages of aggregated data were calculated. A *p*-value equal to or below 5% (*p* ≤ 0.05) was deemed as statistically significant. To provide a ranking of the data related to sources of information, responses were weighted as follows: ‘never’ 0, ‘hardly’ 1, ‘occasionally’ 2, ‘repeatedly’ 3, and ‘continually’ 4. The weighted averages were then calculated and sorted in ascending order. Weighted standard deviations were also determined.

Hundreds of thousands of cross-tabulations were calculated. The large majority of these were non-significant (tested using chi-squared) and when they were statistically significant, effect sizes were very small. For example, we found nothing noteworthy about the effect of respondent age on their responses. Accordingly, we do not report below the results of any cross-tabulations.

When comparing self-reported dietary change frequencies per source, depending on whether participants indicated an increased interest in nutrition since the global pandemic (the ‘yes’ response group) or not (the ‘no’ response group), we ran 220 statistical tests of which 194 were statistically significant at *p* < 0.01 and 16 at 0.01 ≤ *p* ≤ 0.05; only 10 were not statistically significant (*p* > 0.05).

## 3. Results

### 3.1. Misinformed Views about Food, Eating, and Health Practices Related to COVID-19

Table 1 depicts the percentages of respondents who had misinformed views regarding the 25 statements presented to them about certain substances, foods, nutrients, dietary approaches, and health practices in relation to the transmission, infection, protection, or treatment of COVID-19. For example, a large portion (43.1%) of respondents misinformedly agreed that *‘It is safe to eat fruits and vegetables that have been washed with soap or diluted bleach to remove potential COVID-19 viral particles.’* This represents an unfounded or unproven claim of concern and accounts for the largest number of misinformed responses from all 25 statements. Although it was not proven at the time (nor to date) that taking a mega-dose of 12 g of vitamin C daily could help remedy a COVID-19 infection, 27.3% of respondents asserted this to be the case. Vitamin C dosages above 2 to 4 g a day may cause vomiting and other gastrointestinal disturbances [29,30,31], with the added risks of impaired copper and vitamin B12 absorption and iron overload [32].

The two most concerning unfounded or unproven claims (‘gargling bleach’ and ‘ingesting colloidal silver drops’) were deemed as ‘correct’ by 3.3% and 10.5% respondents, respectively. Nearly 4 in 10 respondents misinformedly believed that *‘Antiviral herbs and spices like chilli boost immunity and may help prevent novel coronavirus infection’* (39.7%), that *‘A plant-based diet providing a variety of fruits and vegetables, herbs and spices, wholegrains, legumes, nuts, and seeds can provide immunity against the novel coronavirus and help ‘flatten the curve’’* (39.7%), and that *‘The antiviral properties of garlic and ginger have protective effects against COVID-19’* (39.4%). The statement with the lowest number of misinformed answers corresponds to the false claim that *‘Only people who eat meat are affected by the novel coronavirus’* (1.1%), followed by the precautionary safety measures stemming from official guidelines that *‘To reduce the risk of COVID-19 infection, try to avoid direct contact with the person delivering groceries or packages, and wash your hands thoroughly after bringing in packages or grocery deliveries’* (2%), meaning that the vast majority of the sample were not misinformed in this respect.

### 3.2. Eating Behavior Change Frequency per Information Source

Table 2 shows the frequency of eating behavior changes reported for different sources of information during the early stages of the COVID-19 global outbreak. We found that the extent and frequency of self-reported changes in eating practices depended on the source of information used. The highest proportion of self-reported behavior change was based on information or dietary advice from nutrition scientists, PhDs, and academics (1.53 weighted average), followed by a nutrition professional (1.47), nutrition or health websites (1.33), and scientific journals or science news publications (1.27). On the other hand, the lowest proportion of self-reported eating behavior change was based on information or dietary advice from famous personalities, actors or presenters (0.36), private messages such as WhatsApp, Viber, and Messenger (0.49), and social media such as Facebook or Twitter (0.58).

#### Increased Interest in Nutrition and Subsequent Dietary Change

**Figure 1:**
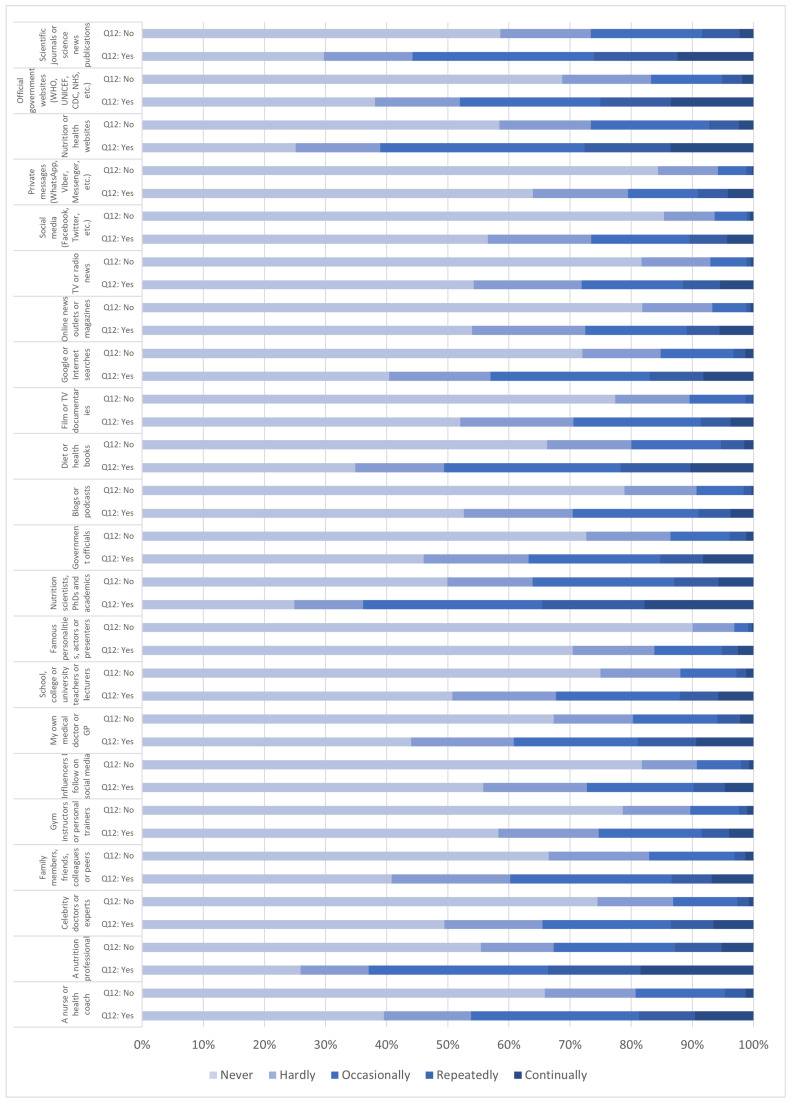
Comparison of dietary change frequencies per source between participants with increased (‘yes’) and no increased (‘no’) interest in nutrition since the start of the COVID-19 pandemic.

Figure 1 shows a comparison of self-reported dietary change frequencies per source, depending on whether participants indicated an increased interest in nutrition since the start of the global pandemic (the ‘yes’ response group) or not (the ‘no’ response group). We observed that participants who indicated an increased interest in nutrition since the global pandemic began reported higher dietary change frequencies based on information from all 22 sources, when compared to those who did not report an increase in interest. For instance, a larger proportion of respondents who became more interested in nutrition stated that they had continually (13.6%), repeatedly (14.0%), or occasionally (33.5%) changed what or how they eat based on information from nutrition or health websites, whereas for respondents whose interest in nutrition did not increase, these changes were markedly less frequent (continually 2.4%, repeatedly 5.0%, occasionally 19.3%; *p* = 0.00). Similarly, the more interested participants also stated that they had more frequently changed their eating as a result of information gained from Google or Internet searches (continually 8.3%, repeatedly 8.7%, occasionally 26.1%), in contrast to those whose interest did not increase (continually 1.4%, repeatedly 1.9%, occasionally 11.9%; *p* = 0.00). These differences were noted across all sources of information used for self-reported dietary change, with overall higher dietary change frequencies revealed by participants who expressed an increased interested in nutrition since the COVID-19 outbreak, as represented in Figure 1. *p* values were calculated when comparing the Never, Hardly, Occasionally, Repeatedly, and Continually response groups against each other for each of the 22 sources of information against each of the other 22 sources of information. We obtained 48,400 *p* values for these 48,400 cross-tabulations. The vast majority were significant at *p* ≤ 0.05.

### 3.3. Proportion of Misinformed Views per Information Source for Dietary Changes

**Figure 2:**
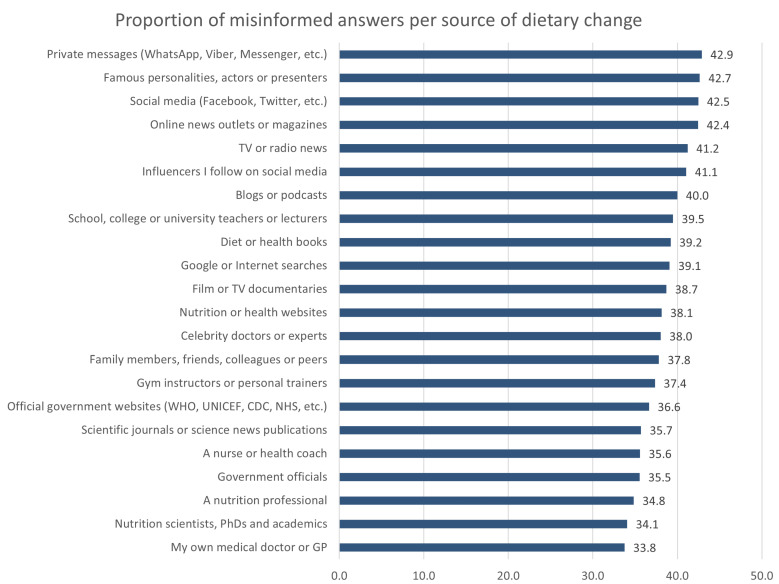
Proportion of COVID-19 nutrition misinformation held in relation to different information sources used for subsequent eating behavior change.

Figure 2 summarizes the relationship between the use of different information sources for changes in eating behavior and the proportion of misinformed views about food, diet, and nutrition concerning COVID-19 during the first-wave multi-national lockdowns. The extent of misinformation held relative to the information sources used for making dietary changes is presented in descending order, from highest to lowest proportion of misinformed views, with the depicted values being weighted averages of misinformed responses. Respondents who self-reported that they had changed what or how they eat based on poorer quality sources gave a larger number of misinformed answers across all 25 statements. For example, those changing their food and eating behavior based on lower quality sources such as private WhatsApp messages, famous personalities, and social media held the greatest proportion of misinformed views (42.9%, 42.7%, 42.5%, respectively). Conversely, we found fewer misinformed views when changing eating behavior was reportedly based on information or advice from respondents’ own medical doctors or GPs (33.8%), from nutrition scientists, PhDs and academics (34.1%), and from nutrition professionals (34.8%). Further information can be found in Supplementary Table S4.

## 4. Discussion

The survey study aimed to capture the respondents’ levels of misinformation by presenting them with interim precautionary guidance statements as well as unfounded or unproven claims surrounding dietary and health practices for COVID-19 prevention or remediation during the origins of the global pandemic, when diagnostic tools, immunization agents, and therapeutics were not yet available [20]. The influence of different sources of information on the frequency of self-reported changes in eating behaviors was also evaluated, alongside the extent of misinformation held depending on the source used for such changes. Additionally, we examined whether an increased interest in nutrition correlated with dietary behavior change frequency.

### 4.1. Magnitude of COVID-19 Nutrition and Health Misinformation Held

Results confirmed widespread misinformation about food, eating, and health practices related to COVID-19 at the start of the first wave, with the 25 statements put to participants receiving between 1.1% and 43.1% misinformed answers. The statement receiving the greatest number of misinformed answers was that it is safe to wash fresh produce with soap or bleach, whereas only a small minority thought that gargling bleach can protect against COVID-19. Our data collection coincided with a May 2020 survey conducted by the U.S. Centers for Disease Control and Prevention (CDC) [13] that found that 19% of respondents intentionally applied bleach to food items (e.g., fruit and vegetables) and 4% gargled diluted bleach and other household cleaning and disinfectant solutions. Cleaning food products with soapy water or diluted bleach, and gargling bleach are hazardous practices that should be avoided as they can lead to poisoning and corrosive tissue injury [33,34,35] with symptoms such as sinus, eye, or skin irritation, upset stomach, nausea, dizziness, headaches, and breathing problems [13]. About one year after our study, a retrospective 12-month global surveillance review [36] reported multiple poisoning events and potentially hazardous practices engaged in the false hope of preventing or treating COVID-19, including the misuse of household cleaning products to sanitize food or to act in lieu of medicine [7].

Other potentially hazardous practices, such as ingesting colloidal silver drops and taking 12 g of vitamin C daily, received low (10.5%) or intermediate (27.3%) misinformation scores, respectively. In March 2020, the U.S. Food and Drug Administration (FDA) and the Federal Trade Commission (FTC) cautioned several companies to stop advertising and selling fraudulent COVID-19 ‘cures’ such as colloidal silver, warning that this bogus product is unsafe and ineffective for treating any ailment [37,38]. The ingestion of colloidal silver can cause irreversible argyria [39] and other adverse effects, including encephalopathy, hepatotoxicity, dysphagia, pulmonary nodules, and metallic layering in the colon [40].

At a time when detection testing and vaccines were not available, the beneficial effects of several nutrients, foods, supplements, and dietary approaches on COVID-19 prevention and treatment were hypothesized in the scientific literature but not yet adequately tested [41,42,43,44,45,46,47], such as the ingestion or intravenous injection of mega doses of vitamins C and D well above recommended thresholds, posing safety concerns [48,49,50,51,52,53,54,55,56]. Nearly 3 in 10 respondents misinformedly affirmed that taking 12 g of vitamin C daily can help remedy a COVID-19 infection, and a smaller proportion (1 in 6) that *‘Taking high-dose vitamin C and D supplements will stop you from catching COVID-19.’* Encouragingly, a majority (8 in 10) recognized the essentiality of vitamin C for immune function (S16). Vitamins C and D, alongside other essential nutrients, can help with the ‘maintenance of functions of the immune system’ (European Food Safety Authority, EFSA) [57]; thus, mitigating deficiency risks for these during the early stages of the novel coronavirus pandemic was emphasized [58,59,60,61,62,63,64,65,66,67,68]. Research on vitamin C and vitamin D status remains ongoing on the grounds that their deficiency may be associated with increased susceptibility to COVID-19 infection and to potentially more severe or prolonged symptoms once infected [69,70,71,72,73,74,75,76,77,78,79,80,81,82]. Because medical prescription is not needed for their procurement, misinformed assurances with silent contraindications about vitamin supplements could lead to harmful intakes, unsafe combinations, and misuse by the public, as seen in hypervitaminosis and intoxication case reports during the global pandemic [36,83,84,85,86,87,88,89].

Misinformation levels for unsubstantiated claims about zinc, green tea, oregano oil, Chaga mushroom blends, cow urine, bear bile, echinacea (S11), and oreganol P73 (S20) were modest (1 in 10 respondents), whereas those concerning garlic and ginger (S7) and herbs and spices such as chili (S9) were high (4 in 10 respondents). A January to April 2020 online content review documented fallacious claims about many of these substances, ingredients, and alleged herbal remedies, in many instances disseminated as ‘recommendations’ to the public [3]. This likely precipitated misinformed beliefs and a potential false sense of security to combat COVID-19, as noted in later accounts around the world associating homeopathic, complementary, and alternative medicine usage or predilection to vaccine hesitancy [90,91,92,93,94] and a greater reluctance to follow official safety guidelines such as social distancing, hand sanitation, and face-mask wearing [95,96]. Cow-urine drinking parties and smearing advice to fight the novel coronavirus were deemed a public health concern in India [97,98], not only due to escalating COVID-19 contagion risk from the resulting large group gatherings, but also because of the potential transmission of infectious agents and diseases from bovine excretions. Approximately 4 in 10 respondents from India (37.7%) gave misinformed answers for statement 11 which contained the cow urine claim, compared to one in 10 (10.6%) respondents from all other countries, suggesting some local popularity of this myth.

Other pseudoscientific practices of a speculative nature to supposedly ‘eliminate’ or ‘neutralize’ the SARS-CoV-2 virus examined in our study included taking vodka sips (S1), sipping hot beverages such as tea and broth (S2), flushing by drinking water (S3), gargling with warm water and added salt, apple cider vinegar, or lemon (S5), gargling with Listerine mouthwash (S6), avoiding cold drinks and cold foods such as ice-cream (S14), and keeping the mouth and throat moist with saliva (S15). Although the number of misinformed views about vodka sips were low (7.4%), those about the rest of these practices were more than two to three times higher. Although most may appear unimportant, regional offices of the World Health Organization aimed to debunk several of these myths, in particular pointing out that alcoholic drinks do not preclude or stop a COVID-19 infection and that harmful use increases health risks [99,100,101,102,103,104,105]. At this time, methanol (disguised as regular alcohol by bootleggers) underlied 5876 hospitalizations and nearly 800 deaths in Iran alone [6,14,36,106].

Rumors about the virus having originated from meat consumption, or that only people who eat meat become affected by it, were included in the survey; however, misinformation levels for these were among the lowest. Precautionary guidance from the World Health Organization issued in February 2020 [107] about avoiding undercooked meat, raw meat, raw milk, and other raw or undercooked animal products, primarily due to the risks of cross-contamination and viral shedding or spillover by food handlers, was also included in our study (S12 and S18), with around a quarter of respondents being misinformed about this. Infectivity risk from SARS-CoV-2 particles on or in meat and animal food products continues to be under investigation and has not yet been entirely ruled out, as alerted by researchers indicating their persistence on imported frozen meat and seafood packages [108,109], on the surface of both frozen and refrigerated meat product samples [110,111], in rare and medium ground beef burgers when cooked [111], and in pig and rabbit cells infected in vitro [112], among others.

The false claim that a varied, whole-foods plant-based diet can ‘provide immunity against COVID-19’ was asserted by almost 4 in 10 respondents. This misbelief coincides with the rise of plant-based eating, veganism, and vegetarianism appeal and interest since the pandemic, with a key motivator being personal health [113,114]. Although research has related certain habitual ways of eating (such as plant-based diets) with nutritional and health advantages and overall disease risk mitigation [115,116], no dietary pattern or approach provides complete protection from COVID-19. The baseless conjectures that alkaline eating can help neutralize SARS-CoV-2 (S22) and that ketogenic eating provides a higher survival chance (S23) received moderate misinformation scores (1 in 6 respondents). Radical dietary changes that exclude entire food groups or that only favor a few foods may result in unwarranted dietary excesses and insufficiencies, compromising overall diet quality and ultimately increasing health risks [117,118,119].

Reassuringly, only very few respondents held misinformed views about adequate physical distancing from delivery personnel and hand-washing after contact with packages and grocery deliveries (S21), suggesting near unanimous recognition of official safety guidance during the first pandemic lockdowns.

### 4.2. Influence of Different Information Sources on Eating Behavior Changes

To our knowledge, no other study has examined the comparative impact of diverse sources of nutrition information and dietary advice on subsequent changes in eating behavior and, more specifically, on their rate of occurrence during the pandemic. Our results show that the influence on eating behavior change frequency across information sources was not uniform. Different sources appeared to result in distinct change frequency effects.

The information sources that resulted in more frequent (‘repeatedly’ and ‘continually’) self-reported eating behavior change were nutrition scientists, PhDs, and academics, and nutrition professionals, closely followed by nutrition or health websites and scientific journals or science news publications. This encouraging finding could signify that there is more trust in these sources given the heavier reliance on them for effecting changes in diet compared to others. However, nutrition information and dietary advice from nutrition or health websites exhibited higher eating behavior change frequencies (‘repeatedly’ and ‘continually’) than those from official government websites (WHO, UNICEF, CDC, NHS, etc.). This is at odds with some research indicating that the latter ranked amongst some of the most used and trusted sources of information during the early stages of the pandemic [120], suggesting that greater source use and trustworthiness may not necessarily result in higher reliance for making changes in eating behavior.

### 4.3. Dietary Changes Based on Information from Lower Quality Sources

Acting on nutrition information and dietary advice from very low-quality sources was less common. The least used sources for changing eating behavior were famous personalities, actors, or presenters, private messages from WhatsApp, Viber, or Messenger, and social media such as Facebook or Twitter. This is a reassuring finding, given that the quality of nutritional information and dietary advice from these sources tends to be poorer and concerningly riskier [121,122,123,124].

Although results showed the greatest reliance on nutrition scientists, PhDs, and academics for eating behavior changes, a slightly higher proportion of respondents reported changing their diet based on information from nutrition or health websites than from scientific journals and science news publications. Plausible explanations for this could include typically lower accessibility to scientific journals, complexity and comprehension challenges, differential keywords or search terms applied, science backlash or growing skepticism, partly caused by rushed pre-prints without peer review [125] and bogus studies indexed in PubMed and Google Scholar [126], a low number of peer-reviewed publications related to diet and nutrition in the context of COVID-19 during the early stages of the pandemic, or that respondents simply sought information from more accessible and easier-to-read websites.

### 4.4. Interest in Nutrition, Online Information Seeking, and Dietary Changes

Respondents who indicated a greater interest in nutrition since the COVID-19 outbreak (56.1%, Supplementary Table S1) reported greater dietary change frequencies using information from all 22 sources compared to those whose interest did not self-reportedly increase. Whether the more interested respondents were actively seeking or passively receiving diet-nutrition information was not explicitly measured in our study. However, these respondents reported changing their eating more frequently out of information sought from Google or Internet searches (continually 8.3%, repeatedly 8.7%, and occasionally 26.1%) than those without any increased interest in nutrition (continually 1.4%, repeatedly 1.9%, occasionally 11.9%; *p* = 0.00). Thus, for participants whose interest in nutrition increased since the global pandemic, dietary changes based on information sought from Google or Internet searches was more common.

The same trend was observed for diet-nutrition information obtained from websites, influencers followed on social media, online news outlets or magazines, blogs or podcasts, and diet or health books, likely representing more extensive information seeking behaviors amongst respondents with a growing interest in nutrition. This partially corresponds with other research correlating diet-nutrition seeking behaviors and subsequent changes in diet ([127,128,129], examples). Furthermore, in our study, the higher incidence of dietary change by the more interested respondents was evident for every diet-nutrition information source used, whether online or offline, both in general and interpersonal contexts (as seen in Figure 1).

Our findings that associate increased interest in nutrition with greater diet-nutrition information seeking and more frequent dietary change complement other data from the same time period evidencing unprecedented search-engine query volumes related to diet and nutrition [130,131], paralleled with record-high food supplement sales [132] and diverging swings in healthier/unhealthier eating practices during home confinements [133,134,135].

### 4.5. Greater Misinformation Susceptibility when Relying on Lower Quality Sources for Changing Diet

Our results reveal a relationship between reliance on information sources of varying quality for making dietary changes and the magnitude of misinformation held about food, diet, nutrition, and health approaches concerning COVID-19. Reliance on lower quality information sources was connected to a greater likelihood of respondents holding misinformed views. In contrast, when reportedly changing diet based on information from better quality sources, the proportion of misinformed views was smaller.

Respondents who demonstrated greater reliance for making dietary changes on poorer quality information sources such as private messages (WhatsApp, Viber, Messenger, etc.), famous personalities, actors or presenters, and social media (Facebook, Twitter, etc.) held more misinformed views across all 25 statements (Figure 2). Aside from these sources being commonly held responsible for spreading misinformation and for amplified misinformation vulnerability [1,15,136,137,138,139,140,141], personal factors such as lower levels of health literacy cannot be discounted [142,143]. Research has indicated that individuals with health literacy deficiencies are more prone to use non-scientific sources such as television, social media, blogs, or celebrity webpages [144,145] and more susceptible to health misinformation [136].

A major concern is how misinformation may detrimentally affect personal decisions related to health, food, and diet, potentially leading to adverse outcomes [136]. The greater influence on eating behavior change from poorer quality sources amongst the most misinformed participants (i.e., those participants who also held more nutrition-health misinformation) suggests disparities in the health risks and safety of the changes performed. If the source used for dietary change contains more hazardous, unsafe, or riskier misinformation, then there is a higher risk of health harm [146]. This underscores the need for educational and communication initiatives to counter misinformation and disinformation exposure and impact, through such approaches as targeted warnings, awareness campaigns, and correction strategies, alongside policy-, technological-, and regulatory-level interventions [1].

Conversely, the lowest nutrition-health misinformation scores were found in respondents who more frequently relied on advice from their own medical doctors, nutrition scientists, PhDs and academics, and nutrition professionals for making dietary changes. This corresponds with other research that highlights that higher trust in medical and health professionals and scientists is associated with a lower susceptibility to COVID-19 misinformation [136,147,148,149].

### 4.6. Strengths and Limitations

One of the strengths of the present study is that it assessed respondents’ views by presenting an assortment of nutrition and health claims to prevent or treat COVID-19 commonly disseminated at the time [3]. The order of the 25 statements was randomly changed for each respondent to preclude order-effect bias [150]. The web-based nature of the study facilitated accessibility and open participation from diverse time zones and countries in strict lockdown, together with rapid data collection at a time when collective understanding of COVID-19 was limited and rapidly changing as a result of emerging research [24]. Information sources continue to evolve and diversify, and the study incorporated 22 of these, including several sub-categories of online and digital information (e.g., Google and Internet searches, WhatsApp messages, online news outlets, social media, scientific journals) as well as interpersonal sources (e.g., medical doctors, nutrition professionals, family and friends, influencers, government officials). The large sample size also strengthened the study, although its geographical, demographic (excluding age), and social diversity is moderate.

Nevertheless, the study carried certain limitations. Firstly, although an opportunity sample has its benefits in terms of easiness of recruitment, no direct generalization of the findings can be made to the wider population; thus, our research can only be deemed as exploratory. That said, the recruited sample was sizeable for this type of research and, although the online survey did not capture qualitative data, no other study has yet been published that simultaneously measures both people’s views about such a broad selection of nutrition and health claims commonly circulated at the time. It is uncertain whether respondents had been exposed to the specific pieces of misinformation beforehand or encountered these for the first time whilst completing the study’s survey. Therefore, conjectures could have been made when discerning the correctness or incorrectness of the 25 statements, although the ‘not sure’ option offered in each instance likely mitigated the issue of binary answer-choice bias (a limitation in health misinformation discernment studies that only offer a binary ‘true/false’ or ‘correct/incorrect’ answer choice). It is possible that the way the statements were written could have biased some responses. For example, there is an inferring truthfulness from the way multiple claims were written, such as the use of logical fallacies (e.g., linguistic non-sequiturs and perlocutory fluency), making the information presented to respondents more easily believable and convincing [12,151,152], thus requiring a more effortful judgement process before disregarding an illusory claim [152,153,154,155].

Another limitation of our study is that it did not measure behavior change specific to the claims and approaches in the statements, but rather unspecified dietary changes influenced by each of the 22 information sources. Furthermore, although eating behavior change frequency per source was measured, the nature, magnitude, and health effects of those changes were not.

Although item non-response bias tends to be a common limitation in online survey studies, whereby skipped questions may interfere with the completeness of the data available for analysis [156], this survey benefited from a high completion rate of 82.6% (i.e., an average of 643 out of 3707 skipped responses per survey question, based on the 13 non-compulsory questions presented after providing consent). Another known limitation of surveys is speeding [157]. However, the average time it took respondents to complete the survey was 7 min and 57 s, suggesting that fast random clicking was relatively uncommon. Searchability bias (searching for answers online before responding) cannot be excluded, yet the average response time indicates that this was less probable, as it would have taken substantially longer to look up information for the many claims, in addition to spending time to answer the rest of the survey.

It is possible that a respondent’s awareness about the general characteristics of the recruited population (i.e., students and subscribers of The Health Sciences Academy) may have set an expectation of health and nutrition evidence-based knowledge and more skepticism towards poorer quality information sources. In our view, this is unlikely to have caused a major response bias because, compared to other methods such as interviews and telephone surveys, anonymous web-based questionnaires, such as that used in our study, are typically accompanied by reduced social desirability bias (i.e., fear of disapproval) and a more honest reporting of sensitive information [158,159,160]. This is supported by results showing the greatest susceptibility to nutrition-health misinformation by respondents who reported relying more heavily on poorer quality information sources for changing how or what they ate, such as social media, instant messaging, and celebrities.

Finally, it worth highlighting that a causal relationship (i.e., misinformation susceptibility misleading dietary decisions and subsequent eating behavior change) cannot be established from our study, but rather whether there was a higher likelihood of misinformation susceptibility (holding more misinformed views) when basing dietary changes on poorer quality information sources.

## 5. Conclusions

The incidence of and susceptibility to falsehoods, unfounded claims, and misinformed food and eating practices that increase the risk of health harm should be prioritized in the debiasing and warnings of misinformation and disinformation and in the dissemination of corrective public health and nutrition messages in future pandemics or any other health emergency events or humanitarian crises.

Appropriate discernment of unfounded or unproven claims was weakest amongst those respondents who more frequently changed eating behavior as a result of information from poor quality sources. High reliance on sources such as social media, WhatsApp messages, and famous personalities for making changes in eating behavior was associated with holding more nutrition-health misinformation. This emphasizes the need for heightened monitoring, rapid corrective measures (e.g., takedowns, effective debunking), and targeted warnings (e.g., education, active pre-bunking), alongside novel counter-measures to tackle poorer quality sources. In contrast, greater reliance on medical and nutrition professionals and scientists for dietary change was associated with reduced misinformation susceptibility, meaning that trust in science should be further encouraged and high-quality nutritional continuing education in the medical and nutrition professions is pivotal.

Increased interest in nutrition since the pandemic began was correlated with more frequent behavioral changes based on information from all sources, whether high- or low-quality, amplifying the odds of misinformed views supporting riskier food, eating, and health decisions. For participants whose interest in nutrition increased, dietary changes based on information sought from Google or other Internet sources was more common than for those whose interest did not increase. Thus, efforts could be placed on trailing more interested online users and engaged audiences through their information-seeking breadcrumbs (digital traces) to mitigate misinformation exposures and further dissemination.

Results showed the highest reliance on nutrition scientists, PhDs, and academics for eating behavior changes, followed by nutrition professionals, nutrition or health websites, and scientific journals or science news publications. The resulting eating change frequencies were nearly threefold for those sources compared to very low-quality sources such as famous personalities, private WhatsApp messages, and social media. This seems likely to signify greater trust in the former sources for guiding self-reported changes in diet, indicating their central role in dietary behavior change and in the dissemination of consistent and responsible nutrition messages that mitigate the risk of health harm.

## Figures and Tables

**Table 1:** Percentage of participants who gave misinformed answers.

Statement		Misinformed Answers	Appropriate Answers	Unsure Answers
**Unfounded or Unproven Claims of Concern**		**% Who Regarded the Statement as being ‘Correct’**	**% Who Regarded the Statement as being ‘Incorrect’**	**% Who Were ‘Not Sure’ about This**
S4	You can protect yourself from the novel coronavirus by gargling bleach.	3.3	89.2	7.5
S8	It has been proven that taking 12 g (12,000 mg) of concentrated vitamin C daily can help remedy a COVID-19 infection.	27.3	40.0	32.7
S13	Ingesting colloidal silver drops can increase the number of immune cells in the body and disintegrate some strains of coronavirus within 12 h.	10.5	42.7	46.8
S19	It is safe to eat fruits and vegetables that have been washed with soap or diluted bleach to remove potential COVID-19 viral particles.	43.1	40.6	16.3
**Other Unfounded or Unproven Claims**		**% Who Regarded the Statement as being ‘Correct’**	**% Who Regarded the Statement as being ‘Incorrect’**	**% Who Were ‘Not Sure’ about This**
S1	Because the alcohol in vodka acts as a steriliser, taking a few sips can kill COVID-19 viruses sitting in the throat.	7.4	69.3	23.3
S2	The COVID-19 virus cannot resist heat and dies when exposed to temperatures above 40 °C (104 °F). Therefore, sipping hot beverages such as tea and broth can help neutralise it.	29.3	48.1	22.7
S3	Drinking water flushes all COVID-19 viral particles into the oesophagus and then the stomach, where they will be completely disintegrated by gastric acid.	21.0	57.1	22.0
S5	Gargling with warm water and salt, apple cider vinegar, or lemon in hot water can eliminate the novel coronavirus from your throat.	27.1	47.3	25.6
S6	Gargling with Listerine mouthwash can help reduce the risk of novel coronavirus infection due to its proven antiviral and antiseptic properties.	17.5	52.6	30.0
S7	The antiviral properties of garlic and ginger have protective effects against COVID-19.	39.4	30.8	29.8
S9	Antiviral herbs and spices such as chilli boost immunity and may help prevent novel coronavirus infection.	39.7	31.7	28.6
S10	Taking high-dose vitamin C and D supplements will stop you from catching COVID-19.	14.7	67.6	17.7
S11	Immune-boosting supplements such as zinc, green tea, oregano oil, Chaga mushroom blends, cow urine, bear bile, and echinacea have been shown to stop a COVID-19 infection.	12.9	60.7	26.5
S14	Cold drinks and cold foods such as ice-cream help the novel coronavirus remain active in your body for longer, so it is important to avoid these.	19.6	59.2	21.2
S15	Keep your mouth and throat always moist, as saliva can encapsulate and deactivate the COVID-19 virus.	24.6	46.2	29.2
S17	It has been shown that the novel coronavirus is foodborne and transmitted through the consumption of meat, even when the meat has been thoroughly cooked.	5.6	75.3	19.2
S20	Oreganol P73, from oregano oil, has a direct killing effect and ability to stop replication of the novel coronavirus in vitro.	11.4	36.2	52.5
S22	Eating non-acidic (i.e., alkaline) foods that have a pH level higher than the novel coronavirus (that is, above 8.5) can help neutralise it.	14.8	47.5	37.7
S23	Ketosis achieved through high-fat, low-carbohydrate ketogenetic eating helps activate immune T-cells in the lungs and provides a higher survival chance against the novel coronavirus than a carbohydrate-loaded diet.	17.2	36.7	46.1
S24	Only people who eat meat are affected by the novel coronavirus.	1.1	93.2	5.7
S25	A plant-based diet providing a variety of fruits and vegetables, herbs and spices, wholegrains, legumes, nuts, and seeds can provide immunity against the novel coronavirus and help ‘flatten the curve’.	39.7	38.7	21.6
**Precautionary Guidance at the Time**		**% Who Regarded the Statement as being ‘Incorrect’**	**% Who Regarded the Statement as being ‘Correct’**	**% Who Were ‘Not Sure’ about This**
S12	Although the risk is extremely low, undercooked meat contaminated with active COVID-19 viral particles could be a potential source of novel coronavirus transmission.	20.9	43.6	35.5
S16	When your daily intake of vitamin C adequately supports your immune system, it might help you recover faster from COVID-19 than if you were deficient in this essential vitamin.	6.9	79.3	13.8
S18	To reduce the risk of transmission of coronaviruses through food, the consumption of raw meat, raw milk, or undercooked animal products should be avoided, especially during the peak of an outbreak.	30.1	47.0	22.9
S21	To reduce the risk of COVID-19 infection, try to avoid direct contact with the person delivering groceries or packages, and wash your hands thoroughly after bringing in packages or grocery deliveries.	2.0	95.8	2.3
**Weighted Average of Answers across All 25 Statements (%)**		**27.2**	**61.5**	**31.1**

**Table 2:** Self-reported frequency of eating behavior change based on different sources of information during the early stages of the global COVID-19 outbreak.

		Frequency of Eating Behavior Changes per Source						
Sources Used for Eating Behavior Changes		Never	Hardly	Occasionally	Repeatedly	Continually	WAVG *	Total Responses *n*
		%						
1	Nutrition scientists, PhDs, and academics	35.9	12.4	26.7	12.5	12.5	1.53	2675
2	A nutrition professional	39.0	11.5	25.2	11.8	12.5	1.47	2680
3	Nutrition or health websites	39.9	14.3	27.2	10.0	8.6	1.33	2684
4	Scientific journals or science news publications	42.6	14.6	24.6	10.3	7.9	1.27	2684
5	Diet or health books	48.7	14.3	22.6	8.0	6.4	1.09	2687
6	Official government websites (WHO, UNICEF, CDC, NHS, etc.)	51.7	14.2	18.0	7.8	8.4	1.07	2685
7	A nurse or health coach	51.1	14.5	21.9	6.6	5.9	1.02	2668
8	My own medical doctor or GP	54.3	15.0	17.6	6.9	6.3	0.96	2673
9	Google or Internet searches	54.4	14.9	19.8	5.7	5.2	0.92	2682
10	Family members, friends, colleagues or peers	52.1	18.1	21.0	4.4	4.4	0.91	2680
11	Government officials	57.7	15.7	16.4	5.1	5.1	0.84	2672
12	Celebrity doctors or experts	60.5	14.4	16.4	4.7	4.0	0.77	2667
13	School, college, or university teachers or lecturers	61.4	15.3	15.4	4.2	3.7	0.73	2665
14	Film or TV documentaries	63.3	15.8	15.6	3.2	2.2	0.65	2679
15	Blogs or podcasts	64.3	15.1	14.9	3.4	2.2	0.64	2673
16	TV or radio news	66.3	14.8	12.0	3.6	3.2	0.63	2666
17	Online news outlets or magazines	66.3	15.3	11.8	3.3	3.3	0.62	2679
18	Influencers I follow on social media	67.2	13.4	12.9	3.5	3.0	0.62	2679
19	Gym instructors or personal trainers	67.2	14.0	13.0	3.1	2.7	0.60	2670
20	Social media (Facebook, Twitter, etc.)	69.2	13.1	11.4	3.7	2.7	0.58	2678
21	Private messages (WhatsApp, Viber, Messenger, etc.)	72.9	13.0	8.5	3.1	2.5	0.49	2677
22	Famous personalities, actors, or presenters	79.1	10.5	7.2	1.8	1.5	0.36	2664

* The ranking of sources used from highest to lowest eating behavior change frequencies is based on weighted averages per source calculated on the scale ‘never’ (0), ‘hardly’ (1), ‘occasionally’ (2), ‘repeatedly’ (3), and ‘continually’ (4).

## Data Availability

Data are available from the corresponding author upon reasonable request.

## Supplementary Materials

The following supporting information can be downloaded at https://www.mdpi.com/article/10.3390/nu15020451/s1, Table S1: General characteristics of the study participants; Table S2: Statements related to COVID-19 used in the survey questionnaire; Table S3: Sources of nutrition information and dietary advice listed in the survey questionnaire; Table S4: Proportion of misinformed answers per source of dietary change. References [57,107,161,162,163,164] are cited in the Supplementary Materials.

## Abbreviations

WAVG—weighted average. SD—standard deviation.
