# Supplementary material for: Susceptibility to COVID-19 Nutrition Misinformation and Eating Behavior Change during Lockdowns: An International Web-Based Survey
Source: Nutrients. 2023 Jan 14;15(2):451. doi: 10.3390/nu15020451 (PMC9861671; doi:10.3390/nu15020451)
Supplement: Supplementary file 1 [file nutrients-15-00451-s001.zip › Supplementary Table S1_MA Ruani and MJ Reiss.pdf]

nutrients

Table S1. General characteristics of the study participants.

	Variables	*n*	%
**Age**	18-20	77	2.3
	21-25	268	7.9
	26-30	364	10.7
	31-35	403	11.8
	36-40	436	12.8
	41-45	381	11.2
	46-50	426	12.5
	51-55	412	12.1
	56-60	280	8.2
	61-65	201	5.9
	66-70	104	3.0
	70 or older	64	1.9
**Gender**	Male	705	20.8
	Female	2,666	78.5
	Other	3	0.1
	Prefer not to say	24	0.7
**Employment status**	Employed, working 40 or more hours per week	929	28.2
	Employed, working 1-39 hours per week	1,232	37.4
	Not employed, looking for work	457	13.9
	Not employed, NOT looking for work	370	11.2
	Retired	240	7.3
	Disabled, not able to work	66	2.0
**Country of residence**	United Kingdom	1,362	40.3
	United States	489	14.5
	India	293	8.7
	Canada	166	4.9
	Australia	134	4.0
	Ireland	88	2.6
	Pakistan	64	1.9
	South Africa	55	1.6
	Egypt	47	1.4

	Variables	*n*	%
	Nigeria	43	1.3
	Spain	34	1.0
	United Arab Emirates	31	0.9
	New Zealand	30	0.9
	Bangladesh	23	0.7
	Philippines	21	0.6
	Ethiopia	18	0.5
	France	18	0.5
	Malaysia	18	0.5
	Kenya	17	0.5
	Netherlands	17	0.5
	Germany	16	0.5
	Singapore	16	0.5
	Italy	15	0.4
	Nepal	15	0.4
	Russian Federation	15	0.4
	Indonesia	12	0.4
	Mexico	11	0.3
	Sweden	11	0.3
	Turkey	11	0.3
	Other 95 countries	293	8.8
**Physical and digital books owned**	0-9	348	10.2
	10-99	1,311	38.4
	100-499	1,213	35.5
	500-999	343	10.0
	1000 or more	202	5.9
**Current diet**	I only eat animal foods	10	0.3
	I only eat plant foods	414	12.2
	I predominantly eat plant foods, plus some animal foods	1,109	32.7
	I predominantly eat animal foods, plus some plant foods	201	5.9
	I eat a balance of plant foods and animal foods	1,658	48.9

	**Variables**	***n***	**%**
**Self-reported health**	Very poor	23	0.9
	Poor	47	1.8
	Somewhat good	365	13.6
	Good	1,285	47.7
	Great	973	36.1
**Increased interest in nutrition since the COVID-19 outbreak**	Yes	1,525	56.1
	No	1,196	44.0
**Being a nutrition professional**	Yes	636	23.4
	No	1,390	51.1
	Studying for it	697	25.6

2
