# Supplementary material for: Susceptibility to COVID-19 Nutrition Misinformation and Eating Behavior Change during Lockdowns: An International Web-Based Survey
Source: Nutrients. 2023 Jan 14;15(2):451. doi: 10.3390/nu15020451 (PMC9861671; doi:10.3390/nu15020451)
Supplement: Supplementary file 1 [file nutrients-15-00451-s001.zip › Supplementary Table S2_MA Ruani and MJ Reiss_edited refs.pdf]

nutrients

MDPI

Table S2. Statements related to COVID-19 used in the survey questionnaire.

Number	Statement	Description
**S1**	Because the alcohol in Vodka acts as a steriliser, taking a few sips can kill COVID-19 viruses sitting in the throat.	Unfounded or unproven claim
**S2**	The COVID-19 virus cannot resist heat and dies when exposed to temperatures above 40°C (104°F). Therefore, sipping hot beverages like tea and broth can help neutralise it.	Unfounded or unproven claim
**S3**	Drinking water flushes all COVID-19 viral particles into the oesophagus and then the stomach, where they will be completely disintegrated by gastric acid.	Unfounded or unproven claim
**S4**	You can protect yourself from the novel coronavirus by gargling bleach.	Unfounded or unproven claim of concern
**S5**	Gargling with warm water and salt, apple cider vinegar, or lemon in hot water can eliminate the novel coronavirus from your throat.	Unfounded or unproven claim
**S6**	Gargling with Listerine mouthwash can help reduce the risk of novel coronavirus infection due to its proven antiviral and antiseptic properties.	Unfounded or unproven claim
**S7**	The antiviral properties of garlic and ginger have protective effects against COVID-19.	Unfounded or unproven claim
**S8**	It has been proven that taking 12 grams (12,000 mg) of concentrated vitamin C daily can help remedy a COVID-19 infection.	Unfounded or unproven claim of concern
**S9**	Antiviral herbs and spices like chilli boost immunity and may help prevent novel coronavirus infection.	Unfounded or unproven claim
**S10**	Taking high-dose vitamin C and D supplements will stop you from catching COVID-19.	Unfounded or unproven claim
**S11**	Immune-boosting supplements like zinc, green tea, oregano oil, Chaga mushroom blends, cow urine, bear bile, and echinacea have been shown to stop a COVID-19 infection.	Unfounded or unproven claim
**S12**	While the risk is extremely low, undercooked meat contaminated with active COVID-19 viral particles could be a potential source of novel coronavirus transmission.	WHO precaution at the time (21 Feb 2020) [107]
**S13**	Ingesting colloidal silver drops can increase the number of immune cells in the body and disintegrate some strains of coronavirus within 12 hours.	Unfounded or unproven claim of concern
**S14**	Cold drinks and cold foods such as ice-cream help the novel coronavirus remain active in your body for longer, so it is important to avoid these.	Unfounded or unproven claim
**S15**	Keep your mouth and throat always moist, as saliva can encapsulate and deactivate the COVID-19 virus.	Unfounded or unproven claim
**S16**	When your daily intake of vitamin C adequately supports your immune system, it might help you recover faster from COVID-19 than if you were deficient in this essential vitamin.	Essentiality of nutrients [57]; Vitamin C status before [161] and during [162] infection

		may play a mitigating role [163]
**S17**	It has been shown that the novel coronavirus is foodborne and transmitted through the consumption of meat, even when the meat has been thoroughly cooked.	Unfounded or unproven claim
**S18**	To reduce the risk of transmission of coronaviruses through food, the consumption of raw meat, raw milk, or undercooked animal products should be avoided, especially during the peak of an outbreak.	WHO precaution at the time (21 Feb 2020) [107]
**S19**	It is safe to eat fruits and vegetables that have been washed with soap or diluted bleach to remove potential COVID-19 viral particles.	Unfounded or unproven claim of concern
**S20**	Oreganol P73, from oregano oil, has a direct killing effect and ability to stop replication of the novel coronavirus in vitro.	Unfounded or unproven claim
**S21**	To reduce the risk of COVID-19 infection, try to avoid direct contact with the person delivering groceries or packages, and wash your hands thoroughly after bringing in packages or grocery deliveries.	WHO precaution at the time (7 April 2020) [164]
**S22**	Eating non-acidic (i.e. alkaline) foods that have a pH level higher than the novel coronavirus (that is, above 8.5) can help neutralise it.	Unfounded or unproven claim
**S23**	Ketosis achieved through high-fat, low-carbohydrate ketogenetic eating helps activate immune T-cells in the lungs and provides a higher survival chance against the novel coronavirus than a carbohydrate-loaded diet.	Unfounded or unproven claim
**S24**	Only people who eat meat are affected by the novel coronavirus.	Unfounded or unproven claim
**S25**	A plant-based diet providing a variety of fruits and vegetables, herbs and spices, wholegrains, legumes, nuts, and seeds can provide immunity against the novel coronavirus and help 'flatten the curve'.	Unfounded or unproven claim

References

57. European Food Safety Authority. Guidance on the Scientific Requirements for Health Claims Related to the Immune System, the Gastrointestinal Tract and Defence against Pathogenic Microorganisms. *EFSA J.* **2016**, *14*, doi:10.2903/J.EFSA.2016.4369.

107. World Health Organization. Coronavirus Disease 2019 (COVID-19): Situation Report, 32. Available online: https://apps.who.int/iris/bitstream/handle/10665/331686/nCoVsitrep02Apr2020-eng.pdf (accessed on 14 December 2022).

161. Ajdžanović, V.; Filipović, B.; Šošić-Jurjević, B.; Miler, M.; Milošević, V. Margins of Beneficial Daily Dosage of Supplements in Prevention of COVID-19. *EXCLI J.* **2021**, *20*, 828, doi:10.17179/EXCLI2021-3790.

162. Lewis, S.L.; Chizmar, L.R.; Liotta, S. COVID-19 and Micronutrient Deficiency Symptoms – Is There Some Overlap? *Clin. Nutr. Espen* **2022**, *48*, 275, doi:10.1016/J.CLNESP.2022.01.036.

163. Gombart, A.F.; Pierre, A.; Maggini, S. A Review of Micronutrients and the Immune System–Working in Harmony to Reduce the Risk of Infection. *Nutr. 2020, Vol. 12, Page 236* **2020**, *12*, 236, doi:10.3390/NU12010236.

164. World Health Organization. COVID-19 and Food Safety: Guidance for Food Businesses. Available online: https://www.who.int/publications/i/item/covid-19-and-food-safety-guidance-for-food-businesses (accessed on 14 December 2022).
