# Supplementary material for: Susceptibility to COVID-19 Nutrition Misinformation and Eating Behavior Change during Lockdowns: An International Web-Based Survey
Source: Nutrients. 2023 Jan 14;15(2):451. doi: 10.3390/nu15020451 (PMC9861671; doi:10.3390/nu15020451)
Supplement: Supplementary file 1 [file nutrients-15-00451-s001.zip › Supplementary Table S3_MA Ruani and MJ Reiss.pdf]

nutrients

MDPI

Table S3. Sources of nutrition information and dietary advice listed in the survey questionnaire.

**Interpersonal Sources of Information**	**General Sources of Information**
A nurse or health coach	Blogs or podcasts
A nutrition professional	Diet or health books
Celebrity doctors or experts	Film or TV documentaries
Family members, friends, colleagues or peers	Google or Internet searches
Gym instructors or personal trainers	Online news outlets or magazines
Influencers I follow on social media	TV or radio news
My own medical doctor or GP	Social media (Facebook, Twitter, etc.)
School, college or university teachers or lecturers	Private messages (WhatsApp, Viber, Messenger, etc.)
Famous personalities, actors or presenters	Nutrition or health websites
Nutrition scientists, PhDs and academics	Official government websites (WHO, UNICEF, CDC, NHS, etc.)
Government officials	Scientific journals or science news publications
