# Supplementary material for: Susceptibility to COVID-19 Nutrition Misinformation and Eating Behavior Change during Lockdowns: An International Web-Based Survey
Source: Nutrients. 2023 Jan 14;15(2):451. doi: 10.3390/nu15020451 (PMC9861671; doi:10.3390/nu15020451)
Supplement: Supplementary file 1 [file nutrients-15-00451-s001.zip › Supplementary Table S4_MA Ruani and MJ Reiss.pdf]

nutrients

MDPI

Table S4. Proportion of misinformed answers per source of dietary change. 1

Misinformed Answers per Source of Dietary Change									
	Sources Used for Eating Behavior Change from Lowest to Highest Proportion of Misinformed Answers	Never	Hardly	Occasionally	Repeatedly	Continually	WAVG*	SD	Total Misinformed Responses on All 25 Statements *n*
				%					
**1**	My own medical doctor or GP	24.7	27.1	29.1	33.4	41.2	33.8	1.4	12,728
**2**	Nutrition scientists, PhDs and academics	22.3	25.6	27.2	32.5	41.1	34.1	1.4	12,724
**3**	A nutrition professional	22.4	26.6	28.1	33.4	41.3	34.8	1.4	12,747
**4**	Government officials	25.1	24.9	31.6	34.4	44.8	35.5	1.6	12,746
**5**	A nurse or health coach	23.2	27.7	30.7	37.4	43.5	35.6	1.5	12,687
**6**	Scientific journals or science news publications	22.5	26.8	29.9	35.1	44.4	35.7	1.5	12,789
**7**	Official government websites (WHO, UNICEF, CDC, NHS, etc.)	23.9	23.9	29.9	33.7	45.0	36.6	1.5	12,805
**8**	Gym instructors or personal trainers	24.6	30.3	32.5	38.1	49.2	37.4	1.7	12,693
**9**	Family members, friends, colleagues or peers	23.4	27.4	32.7	41.9	48.6	37.8	1.8	12,777
**10**	Celebrity doctors or experts	23.8	30.0	30.9	40.0	49.4	38.0	1.8	12,679
**11**	Nutrition or health websites	21.5	25.6	30.4	37.7	48.3	38.1	1.7	12,792
**12**	Film or TV documentaries	23.6	30.4	36.0	42.3	50.1	38.7	1.7	12,766

**13**	Google or Internet searches	22.7	29.3	33.1	43.7	47.3	39.1	1.6	12,782
**14**	Diet or health books	22.4	27.5	31.3	39.7	50.5	39.2	1.8	12,792
**15**	School, college or university teachers or lecturers	23.5	28.4	34.5	43.6	49.1	39.5	1.7	12,671
**16**	Blogs or podcasts	23.3	30.7	38.1	41.1	51.6	40.0	1.6	12,792
**17**	Influencers I follow on social media	23.9	30.4	35.9	46.9	50.9	41.1	1.8	12,774
**18**	TV or radio news	23.9	29.9	36.1	44.0	52.6	41.2	1.9	12,681
**19**	Online news outlets or magazines	23.9	30.7	36.3	47.0	53.6	42.4	1.9	12,766
**20**	Social media (Facebook, Twitter, etc.)	23.7	31.9	37.8	46.6	52.5	42.5	1.7	12,756
**21**	Famous personalities, actors or presenters	25.0	32.7	38.4	46.8	55.4	42.7	1.9	12,653
**22**	Private messages (WhatsApp, Viber, Messenger, etc.)	24.0	33.6	38.8	47.7	51.1	42.9	1.7	12,755

* The ranking of sources used for eating behavior change from lowest to highest proportion of misinformed answers is based on weighted averages of frequencies per source calculated on the scale 'never' (0), 'hardly' (1), 'occasionally' (2), 'repeatedly' (3), and 'continually' (4).
